# Greenhouse conditions induce mineralogical changes and dolomite accumulation in coralline algae on tropical reefs

**DOI:** 10.1038/ncomms4310

**Published:** 2014-02-12

**Authors:** Guillermo Diaz-Pulido, Merinda C. Nash, Kenneth R.N. Anthony, Dorothea Bender, Bradley N. Opdyke, Catalina Reyes-Nivia, Ulrike Troitzsch

**Affiliations:** 1 Griffith School of Environment, Australian Rivers Institute—Coast and Estuaries, and Australian Research Council Centre of Excellence for Coral Reef Studies, Nathan Campus, Griffith University, 170 Kessels Road, Brisbane, Nathan, Queensland 4111, Australia; 2 Research School of Physics, The Australian National University, Acton, Australian Capital Territory 0200, Australia; 3 Australian Institute of Marine Science, Townsville, Queensland 4810, Australia; 4 Global Change Institute and Australian Research Council Centre of Excellence for Coral Reef Studies, The University of Queensland, St Lucia, Brisbane, Queensland 4072, Australia; 5 Research School of Earth Sciences, The Australian National University, Acton, Australian Capital Territory 0200, Australia

## Abstract

Human-induced ocean acidification and warming alter seawater carbonate chemistry reducing the calcification of reef-building crustose coralline algae (CCA), which has implications for reef stability. However, due to the presence of multiple carbonate minerals with different solubilities in seawater, the algal mineralogical responses to changes in carbonate chemistry are poorly understood. Here we demonstrate a 200% increase in dolomite concentration in living CCA under greenhouse conditions of high pCO_2_ (1,225 μatm) and warming (30 °C). Aragonite, in contrast, increases with lower pCO_2_ (296 μatm) and low temperature (28 °C). Mineral changes in the surface pigmented skeleton are minor and dolomite and aragonite formation largely occurs in the white crust beneath. Dissolution of high-Mg-calcite and particularly the erosive activities of endolithic algae living inside skeletons play key roles in concentrating dolomite in greenhouse treatments. As oceans acidify and warm in the future, the relative abundance of dolomite in CCA will increase.

## Abstract (web summary)

Ocean acidification is affecting the stability of coral reefs, but the exact mineralogical response is poorly understood. Diaz-Pulido *et al.* show that, under warming conditions, the relative abundance of dolomite increases by as much as 200% and could therefore slow the climate-induced break-up of coral reefs.

## Graphical abstract

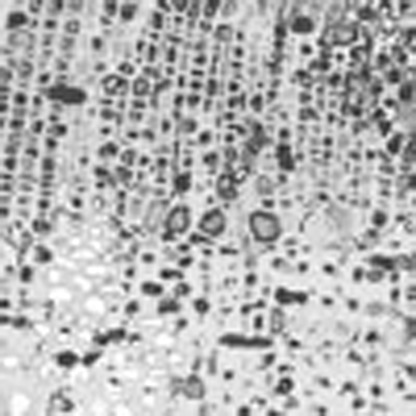

Crustose coralline algae (CCA) are calcifying red algae that are particularly abundant in tropical shallow reefs and are important coral reef builders^1^,^2^. The skeletons of CCA are composed primarily of highly soluble high-Mg-calcite in the cell walls, but also contain cell and pore infill by the more stable carbonates, aragonite^3^,^4^ and dolomite^5^. It has been proposed that the carbonate mineralogy of marine calcifying organisms has varied throughout geologic history, primarily due to oscillations in the Mg:Ca ratio of seawater^6^,^7^,^8^. For example, reduction of the seawater Mg:Ca ratio from >2 (‘aragonite and high-Mg-calcite seas’) to <2 (‘calcite and dolomite seas’)^7^,^9^ caused a shift in mineralogy from high-Mg to low-Mg-calcite in newly formed skeletons of living articulate^7^ and CCA^8^, and from aragonite to calcite in coral skeletons^9^. Increases in atmospheric pCO_2_ and an associated decline in seawater pH during greenhouse conditions also affect carbonate mineralogy^10^,^11^, for instance, by reducing the proportion of aragonite compared with calcite in shells of bi-mineralic marine invertebrates and promoting the precipitation of low-Mg-calcite^11^,^12^. Dolomite, abundant in the geological record, but relatively rare in shallow modern marine environments^13^,^14^, is also positively correlated with greenhouse periods of elevated CO_2_, temperatures, sea level and ‘calcite seas’^15^,^16^. However, prior to the present study, there has been no direct experimental evidence demonstrating the influence of seawater pCO_2_ and/or temperature on concentrations of biomineralized (*sensu*^5^) dolomite^17^,^18^ and whether this influence can drive change without a shift in Mg:Ca.

The presence of different carbonate forms in CCA skeletons provides a unique system to test how the relative abundances of dolomite, aragonite and high-Mg-calcite may vary in response to changes in pCO_2_. CCA with high-Mg-calcite skeletons are predicted to be highly susceptible to skeletal dissolution under human-induced ocean acidification (high pCO_2_ and low pH) scenarios^19^,^20^,^21^. On the other hand, recent work found that dead skeletal fragments of dolomite-rich CCA have lower rates of dissolution than predominantly high-Mg-calcite CCA, and that this type of dolomite is common in shallow tropical coral reefs, particularly in high energy wave exposed habitats^22^. Understanding how the carbonate mineralogy of CCA changes under greenhouse conditions of high pCO_2_ and warming will provide insight into the physical stability of coral reefs under ocean acidification, particularly in shallow habitats and algal ridges where CCA cementation plays a key role in maintaining reef structural integrity^23^.

The mineralogical responses of calcifying organisms to fluctuations in seawater carbonate chemistry and temperature are also influenced by the degree of skeletal protection provided by the living (organic) tissue^24^,^25^. Recent experiments suggest that the presence of protective organic layers is critical in reducing skeletal dissolution of calcifying invertebrates^25^,^26^. Further, photosynthetic activity of some corals and coralline algae increases pH and modifies carbonate chemistry internally (that is, at their site of calcification^27^) and at their surface (that is, within the diffusion boundary layers^28^), with implications for their calcification responses to ocean acidification. In reef-building CCA, the photosynthetically active organic tissue is generally restricted to the upper surface (cortex), while below, the skeleton is devoid of pigmented tissue. Despite the importance of the living tissue in regulating the calcification process in calcifying organisms and modulating responses to ocean acidification^25^,^26^,^29^, the role of the living and photosynthetically active CCA tissue in preventing mineralogical changes is unknown.

Here we address two key questions through experimental manipulations of seawater CO_2_ concentrations and temperature on the Great Barrier Reef (Australia). First, we ask how the carbonate mineralogy of *Porolithon onkodes*, one of the most abundant reef-building CCA^1^ changes in response to ocean acidification and warming (greenhouse conditions). Here, analyses test whether the relative abundance of dolomite in living CCA is favoured under greenhouse conditions (see Methods for mineralogy analyses and Supplementary Table 1 for carbonate chemistry parameters). Second, we explore the extent to which the pigmented (surface living) algal tissue governs mineralogical changes due to ocean acidification. The surface (~ upper 100–500 μm) pink layers of *P. onkodes* tissue are photosynthetically active and are the sites for skeletal deposition^30^. Underneath those layers, the skeleton is white and lacks CCA photosynthetic activity (see Methods). Endolithic algae (mainly cyanobacteria) are typically present below the pigmented CCA tissue and in the basal layers of the white crust, and play an active role in bioerosion^31^. We demonstrate that the abundance of dolomite in living CCA increases by as much as 200% in 8 weeks under a combination of high seawater CO_2_ (1,225 μatm) and temperature (30 °C) treatments. These changes occur mainly in the unpigmented CCA skeleton, suggesting a strong control of photosynthetic algal tissue over skeletal mineralogy. We propose that the relative increase in dolomite is largely due to endolithic algal activity removing calcium from the surrounding high-Mg-calcite skeleton, while the more resistant dolomite mineral is left intact. Taken together, our study shows that dolomite accumulation in a dominant reef-building organism is driven by greenhouse environmental conditions and mediated by biological mechanisms via bioerosion by endolithic algae.

## Results

### Mineralogical changes

**Figure 1:**
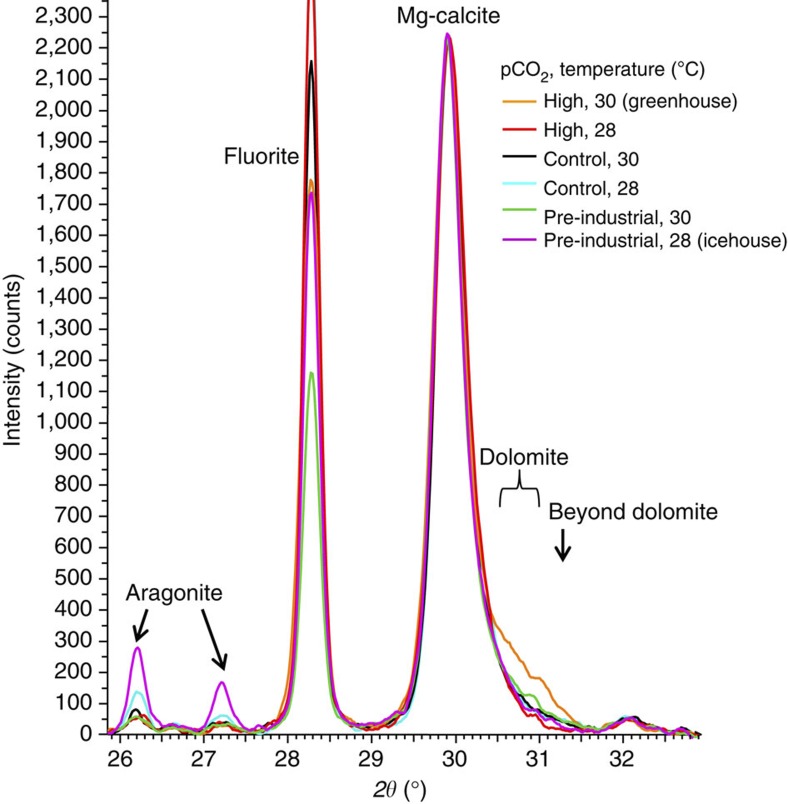
Mineral changes in coralline algae induced by pCO_2_ and temperature. XRD scans for each pCO_2_ and temperature treatment have been added to give a combined average. The more pronounced asymmetry over the dolomite position for the greenhouse condition (orange line) is larger than that of the other scans. This equates to a relative increase in asymmetry of 88% (Fig. 2a) (Methods) and includes three identifiable higher Mg-carbonate phases: High Mg-calcite (H-HMC~26–32 mol% MgCO_3_), dolomite (~38–54 mol% MgCO_3_) and a higher Mg-dolomite approaching magnesite (~95 mol% MgCO_3_). The aragonite peak is highest for the pre-industrial and ambient temperature (28 °C) treatment (purple line).

**Figure 2:**
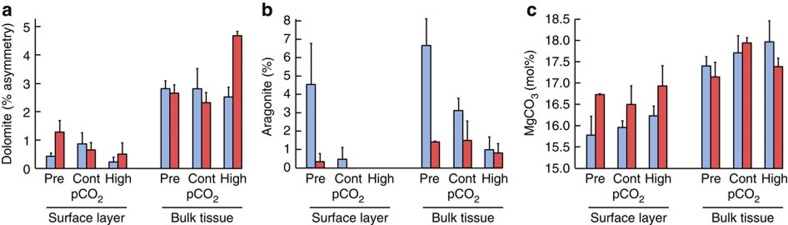
Influence of photosynthetic algal tissue and pCO_2_ and temperature on carbonate mineralogy. Photosynthetically active tissue (surface layer) governs the mineralogical response (that is, dolomite (**a**), aragonite (**b**) or MgCO_3_ (**c**)) of crustose coralline algae when exposed to pre-industrial (Pre), control (Cont) and High pCO_2_ concentrations and ambient (28 °C, blue bars) and warming (30 °C, red bars) temperatures. (**a**) Total asymmetry as an indicator of dolomite amount is higher in bulk tissue (ANOVA, *P*<0.001, *n*=3) than the surface layer and asymmetry increased under greenhouse conditions (high pCO_2_ and 30 °C) only in bulk samples (*P*<0.001, *n*=3). (**b**) Aragonite % is higher in bulk samples (*P*<0.001, *n*=3) and under pre-industrial pCO_2_ and ambient temperature (28 °C) (*P*=0.001, *n*=3). Negligible amounts were found in greenhouse surface layer samples. (**c**) High- MgCO_3_ (mol% MgCO_3_) has less Mg-content in surface layers (*P*<0.001) than in the bulk tissue, but an increase in surface layers is observed under warming conditions (*P*=0.004, *n*=3). Data are presented as means and error bars denote s.e.m.

The mineral dolomite was present in all samples examined with X-ray diffraction (XRD). However, dolomite content in CCA exposed to high pCO_2_ (1,225 μatm) and high temperature (30 °C) was dramatically higher than in CCA exposed to high pCO_2_ and ambient temperature (28 °C) (Fig. 1). A two-fold increase in % asymmetry (an expression of the extent of asymmetry on the XRD Mg-calcite peak and indicator of dolomite content, see Methods) occurred in CCA under greenhouse conditions, compared with CCA under high CO_2_, but only 28 °C (Fig. 2a, analysis of variance (ANOVA), *P*<0.001, see Supplementary Table 2 for ANOVAs). Similarly, a threefold (200%) increase in the relative quantity of dolomite (weight % of total carbonate) occurred under greenhouse conditions (mean 3.8%, s.d. 0.85% (at 28 °C) compared with 11.4%, SD 1.61% (at 30 °C); Tukey HSD multiple comparison, *P*=0.002, Supplementary Tables 3 and 4 for raw data, and Supplementary Table 5 for ANOVAs). Thus, at elevated CO_2_, only a small change in temperature is required to initiate a mineralogy shift. In contrast, the aragonite content in CCA was increased in cool non-acidified water (Figs 1 and 2b, Tukey HSD multiple comparison, *P*=0.001, Supplementary Table 2 for ANOVAs), with 6.6 weight % (s.d. 1.95%) in the pre-industrial CO_2_ concentration (296 μatm, 28 °C) and ~3.1% (s.d. 0.87%) in ambient control CO_2_ (28 °C), compared with <1.5% in the remainder treatments (Supplementary Tables 4 and 5 for ANOVAs). These results demonstrate that interactions between pCO_2_ and temperature are critical determinants of mineralogical changes in coralline algae.

### Role of pigmented tissue in CCA mineralogy

**Figure 3:**
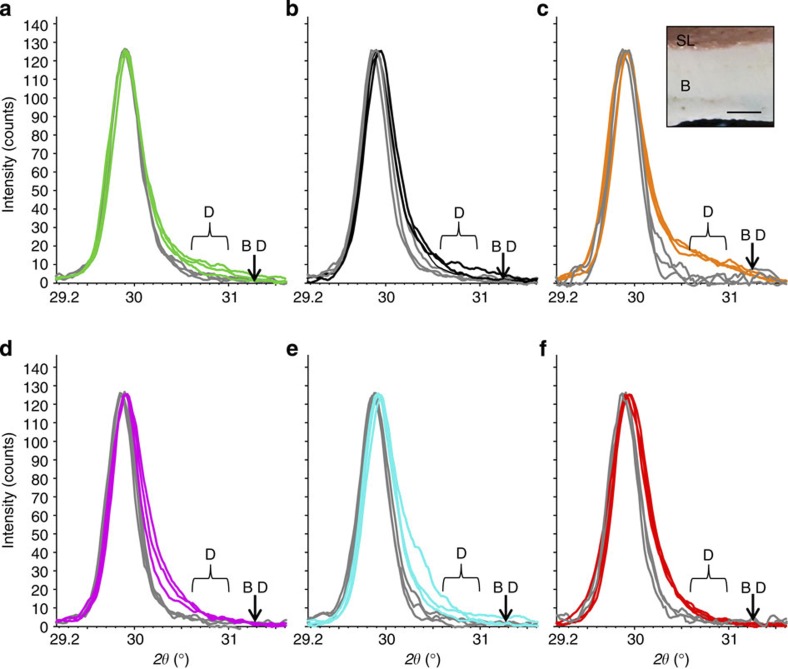
Comparisons of skeletal mineralogy between photosynthetically active tissue (surface layer) and bulk tissue. XRD scans for surface scrapings of pigmented coralline skeleton (grey lines) and bulk skeletons (coloured lines) across pCO_2_ and temperature treatments. (**a**) Pre-industrial pCO_2_, 30 °C, (**b**) control pCO_2_, 30 °C, (**c**) high pCO_2_, 30 °C, (**d**) pre-industrial pCO_2_, 28 °C, (**e**) control pCO_2_, 28 °C and (**f**) high pCO_2_, 28 °C. Scans for the surface scrapings are less asymmetrical than those of the bulk skeletons, indicating that the Mg-carbonate is all, or predominantly, Mg-calcite. Inset shows cross-section of CCA with surficial photosynthetically active tissue layer (SL) and nonpigmented skeleton (bulk sample, B); scale bar, 2 mm. D: dolomite range; BD: beyond dolomite.

To test the significance of the living (pigmented) CCA tissue in governing the mineralogical response, we compared the XRD scans of the surficial skeleton (surface scrapings of the pink layer) with those of the bulk sample skeletons. XRD scans for the bulk samples typically displayed strong Mg-calcite peak asymmetry indicating dolomite presence^5^, whereas peaks for the surficial skeleton were less asymmetrical indicating the Mg-carbonate is entirely, or predominantly, high-Mg-calcite (Fig. 3). Importantly, we found dolomite mainly in the skeleton lacking pigmented algal tissue. Aragonite, similar to dolomite, was more abundant in the unpigmented skeleton compared with the surface scrapings. Increased pCO_2_ did not affect the mol% MgCO_3_ of the pigmented Mg-calcite skeleton, suggesting that there is no skeletal plasticity associated with elevated pCO_2_ over the course of the experiment, which is in contrast to recent mineralogical studies with branching coralline algae^12^,^32^ that showed a reduction in the % mol MgCO_3_ under ocean acidification. Increased temperature in our study, however, facilitated the incorporation of Mg in the skeleton covered by photosynthetic tissue, with Mg-content increasing from 15.99 mol% MgCO_3_ (mean across CO_2_ levels, s.d. 0.39%) at 28 °C to 16.73 mol% MgCO_3_ at 30 °C (s.d. 0.46%, Fig. 2c; ANOVA, *P*<0.001, Supplementary Table 2 for ANOVA), in line with previous studies of temperature effects on temperate CCA^33^. These results suggest that live, pigmented CCA tissues limit mineralogical shifts from high-Mg-calcite to dolomite, and suppress changes in Mg incorporation due to elevated pCO_2_, but cannot compensate for temperature influence.

### Presence of dolomite and aragonite in CCA

**Figure 4:**
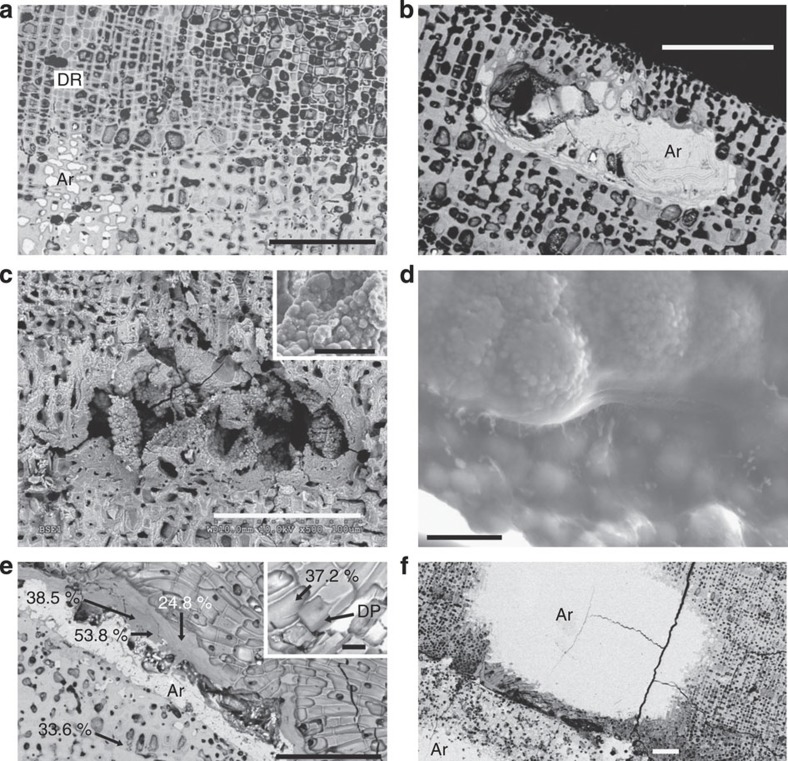
Dolomite and aragonite in crustose coralline algae (CCA). (**a**) CCA from greenhouse conditions showing numerous dolomite rims (DR, grey). The aragonite (Ar) in-filled cells with dolomite rims appear in small patches throughout the sample. The bottom half of the image has thicker cell walls that may reflect winter growth^60^. (**b**) Conceptacle in-filled with aragonite (Ar) in the pre-industrial, ambient temperature sample. (**c**) Old, buried conceptacle in-filled by spheroidal dolomite (greenhouse sample); the inset is a close-up of the internal conceptacle surface, suggesting possible microbial origin. (**d**) Spheroidal (possibly microbial) dolomite inside former conceptacle (pre-industrial sample). (**e**) Dolomite (grey) and aragonite (white) cement-like alteration of parallel bands in the bulk skeleton. Remnants of the original cell fabrics are preserved in both dolomite and aragonite bands. Values indicate mol% MgCO_3_. Inset showing dolomite pod (DP) (greenhouse sample). (**f**) Aragonite (Ar) alteration pervasively replaced Mg-calcite and dolomite in pre-industrial treatment. Scale bars, 100 μm (**a**); 100 μm (**b**); 100 μm (**c**), 20 μm (inset); 5  μm (**d**); 100 μm (**e**), 20 μm (inset); and 100 μm (**f**).

To understand how the additional dolomite and aragonite were present in the CCA, selected samples from the different pCO_2_ and temperature treatments were examined by scanning electron microscope-energy dispersive spectroscopy (SEM-EDS, Methods). CCA across the treatments typically had dolomite cell lining, aragonite cell infill and dolomite and aragonite conceptacle infill (Fig. 4a–c) similar to features documented previously^3^,^5^,^22^. The morphology of the dolomite infill in conceptacles and some cell spaces (Fig. 4d) is comparable with spheroids and dumbbells imaged for microbial^14^,^34^ and synthetic dolomite^35^. Dolomite and aragonite were also present as cement-like alteration of parallel bands of the bulk skeleton (Fig. 4e). The extra aragonite in pre-industrial treatment seems to be present as more cell infill and larger areas of skeletal alteration (Fig. 4f). In the high CO_2_ and high temperature CCA, the bands of dolomite had higher Mg-content tending towards stoichiometric dolomite, whereas in pre-industrial treatments, they were calcium enriched.

### Increase of dolomite and role of endolithic algae

**Figure 5:**
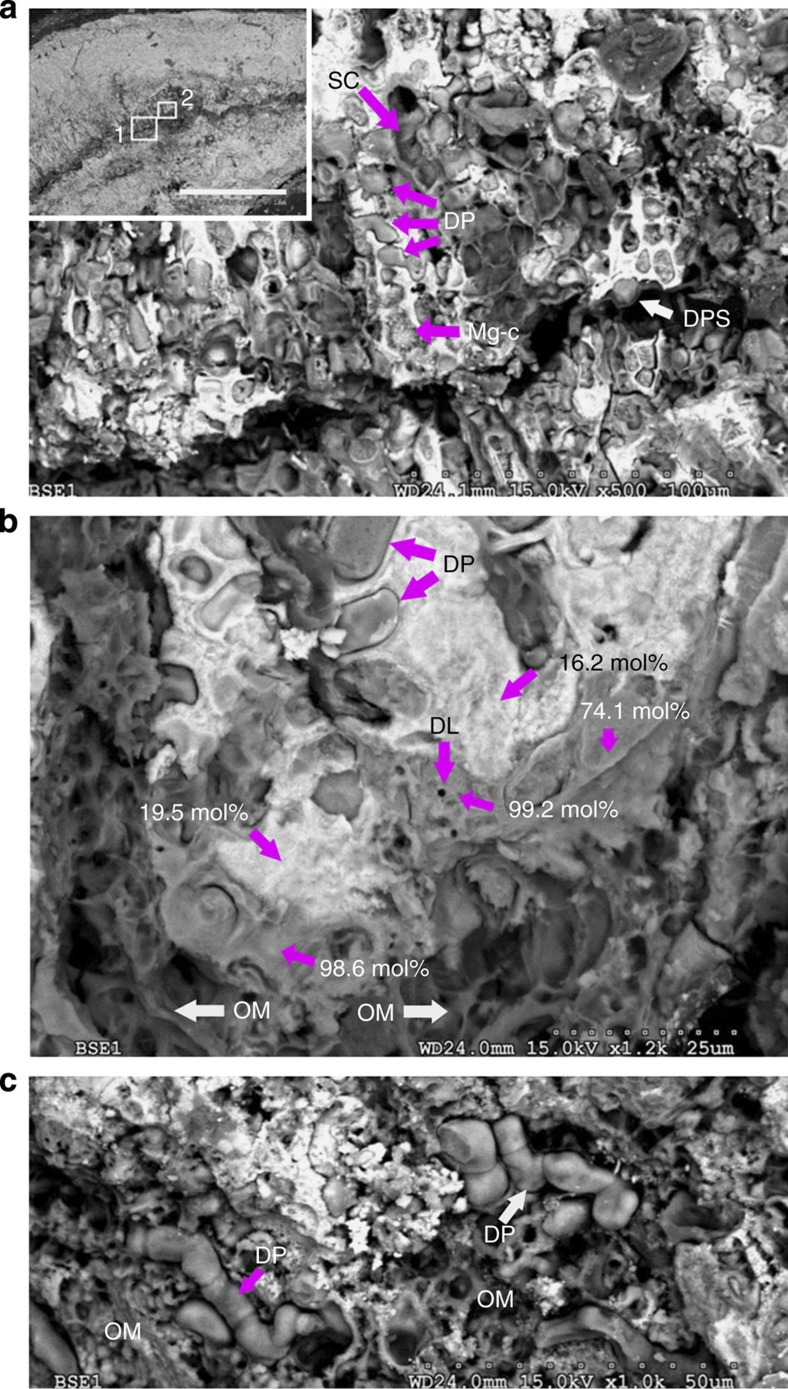
Process of relative dolomite enrichment in crustose coralline algae in greenhouse treatments. (**a**) Box 1 of inset: Dissolution of Mg-calcite along internal band. Enhanced microborer activity^21^ and likely penetration of undersaturated seawater with respect to high-Mg calcite dissolves crustose coralline algae (CCA) skeletons exposing dolomite pods—dolomite casts of cells (DP). SC: String of CCA cells has been removed leaving empty space behind. Dolomite pod suspended (DPS) on organic matrix. (**b**) Box 2 of inset in (**a**), top and middle of the image shows removal of interfilament Mg-calcite exposing dolomite pods; some cell wall high-Mg calcite remains (whitish areas, for example, 16.2 and 19.5 mol%) and cell features are visible. Dolomite lining (DL). Calcium is being removed from the skeleton leaving mainly the Mg-carbonate (for example, 74.1, 98.6 and 99.2 mol%). The bottom of the image shows removal of carbonates leaving only the organic matrix (OM). (**c**) Removal of Mg-calcite exposes connected dolomitized pods (DP). Scale bar, 1 mm (**a**).

The increase in relative dolomite content under greenhouse conditions was easily visible in the unpigmented CCA skeleton, seen as an obvious endolithic algal band (Fig. 5a–c). Here, dissolution of high-Mg-calcite leaves behind Mg-enriched remnants and exposed dolomite features. It appears the skeletal break-up takes place by removal of calcium from the Mg-calcite skeleton leaving up to near-magnesite (for example, >98 mol% MgCO_3_) composition and finally, complete dissolution of this Mg-rich remnant skeleton (Fig. 5b). Where the Mg-calcite cell wall has been removed, connected dolomite cells remain following the coralline algal filament morphology (for example, sausage-like) and decalcified algal organic matrix (Fig. 5c).

We have previously noted an increased abundance of endolithic algae under greenhouse conditions, which was correlated with weight loss of CCA^21^. Here we examined the relationship between the skeletal dissolution (that is, weight loss) and dolomite abundance (asymmetry %) and found a positive relationship in bulk skeletons under the high temperature treatment (*r*^2^=0.48; *P*=0.039; *n*=9, Supplementary Fig. 1). Further, not surprisingly^19^,^20^,^21^,^36^, enhanced pCO_2_ caused increased dissolution of algal skeletons, a trend that was exacerbated by elevated temperature (2-way ANOVA, *n*=90, CO_2_: *F*=24.2, *df*=2, *P*<0.001; temperature: *F*=8.63, *df*=1, *P*=0.004). These results support the observation that skeletal dissolution associated with endolithic algal activity contributes to increased dolomite concentration in CCA, although the increase observed here may be more than that would occur in the natural environment where the CCA sides are not fully exposed (*cf*. ref. 37).

## Discussion

Our experimental results demonstrate that interactions between elevated seawater pCO_2_ and warming drive mineralogical changes in reef-building coralline algae. These changes, however, depended on the type of carbonate mineral analysed and presence of photosynthetically active (pigmented) CCA tissue. Increasing seawater CO_2_ concentrations alone did not lead to large mineralogical changes, but a temperature increase of just two degrees (28 °C up to 30 °C) in the high CO_2_ treatment, led to a significant increase (~200%) in the relative amount of the carbonate dolomite in the experimental coralline algae. The relationship between the abundance of dolomite in the shallow marine sedimentary record and greenhouse conditions is documented in the literature^15^,^16^,^38^. This relationship may be due to high atmospheric CO_2_ levels and/or warmer temperatures, or because of global high sea level during greenhouse conditions leading to an expansion of warm shallow seas (epeiric seas) that are more conducive to dolomite formation^15^,^38^,^39^,^40^, or a combination of all factors. However, our study provides unique experimental evidence of the critical importance of the combination of high pCO_2_ and high sea surface temperature, and biological processes by endolithic cyanobacteria in the concentration of biomineralized dolomite in the coralline alga *P. onkodes*. Since *Porolithon* species are dominant and major framework builders in Pacific and Caribbean shallow reefs^1^,^23^, these results may have implications for understanding dolomite accumulation and distribution in the marine environment.

The presence of pigmented (photosynthetically active) tissue layers played an important role in modulating the mineralogical responses of the coralline alga *P. onkodes* to increased pCO_2_/reduced pH and temperature. Dolomitisation was mainly observed in the unpigmented, non photosynthetic CCA tissue (Figs 2 and 3). The mechanisms for this are not clearly understood, but may be related to particular types of organic substrates produced by the unpigmented layers. Recent research has shown that organic matter from other red algae^41^,^42^ and microbial biomass^34^ containing high carboxyl groups provides the substrate needed for dolomite nucleation^43^, and no concurrent living (metabolic) processes are required for this nucleation (that is, dolomite forms best when the organism is dead^43^). Pigmented tissue likely involves prevention of colonization and subsequent bioerosion of internal skeleton by endolithic cyanobacteria, as cyanobacteria are mainly observed in the white crust beneath the pink tissue. Pigmented tissues may also avoid mineral changes by protecting the CCA skeleton from surrounding undersaturated seawater (for example, Ries^12^), and/or by regulating internal metabolic chemistry (for example, pH and carbonate saturation state of the calcifying medium as suggested for a range of marine calcifiers^25^,^26^,^27^,^44^).

We propose two possible hypotheses explaining the increased dolomite abundance found in our experiments: first, that additional dolomite has formed, and second, that only the relative dolomite abundance has increased due to dissolution of Mg-calcite skeleton. Examining the first hypothesis, we need to consider the two types of dolomite present in CCA; (1) primary dolomite (that is, conceptacle infill and cell lining and infill), and (2) secondary (alteration) dolomite (that is, relative Mg enrichment as a result of calcium removal by endolithic algal activity). Considering the primary dolomite or cell lining dolomite: this seems to form with a consistent thickness constrained by cell morphology and there was no detectable difference between treatments. The infill by spheroidal dolomite that appears to form in or on an organic matrix (Fig. 4c,d) was more easily detectable in CCA under greenhouse conditions. The spheroidal dolomite seems the most likely dolomite to respond to temperature as the spheroidal morphology is very similar to microbially associated dolomite^14^,^34^ and microbial activity and abundance in CCA can increase with temperature^21^,^45^. Alternatively, as dolomite can form abiotically requiring only sufficient concentration of red algae agar organic substrate^41^, then this type of dolomite may also increase as agar production can rise with higher seawater temperature^46^. Considering the secondary dolomite: alteration bands of dolomite cement were observed more commonly in the CCA from the greenhouse treatment compared with the icehouse, and this appears a result of cyanobacterial activity, which increases in greenhouse conditions, and Mg-calcite skeleton with calcium partially removed was measured by SEM-EDS to have compositions in the range of dolomite approaching magnesite.

The second hypothesis of relative dolomite increase is supported by two facts, our previous research documenting the increase in endolithic algal activity with ocean acidification and warming^21^,^47^, together with the visible evidence of endolithic algal colonisation in CCA under greenhouse conditions leading to extensive localized high-Mg-calcite destruction, while dolomite cells remain intact (Fig. 5c). Thermodynamically, Mg would be expected to be lost from the skeleton under greenhouse conditions, likely due to chemical dissolution caused by undersaturation of seawater with respect to high-Mg-calcite^48^ (Supplementary Table 1), potentially favouring accumulation of less soluble calcium carbonate forms such as dolomite. However, endolithic cyanobacteria that are common in CCA (for example, *Mastigocoleus* sp.^31^) have been documented to actively remove calcium from calcitic skeletons^49^. As calcium removal is only observed in the endolithic band, this suggests that it is the endolithic cyanobacteria that are removing calcium, leading to the destruction of the Mg-calcite skeleton while the dolomite components remain intact (Fig. 5). Recent studies show that *Mastigocoleus* easily bores calcitic and aragonitic substrates, but is unable to bore into dolomite^50^. Thus, a relative accumulation of dolomite is also a reasonable explanation for its increased abundance in the greenhouse samples. Here we have demonstrated the role of endolithic algae in producing a relative increase in dolomite, to the formation of dolomite and to higher Mg-rich phases by calcium removal, and speculated about an increase in the formation of cell lining and infill dolomite. In the end, and possibly most likely, the increased concentration of dolomite in CCA under greenhouse environments could be caused by a combination of processes from both hypotheses. We believe the process observed here is unlikely to be restricted to the coralline alga *P. onkodes* as endolithic cyanobacterial erosion is common in other marine carbonates, not just CCA. It may be possible that dolomite could form by default in other Mg-calcite skeletons or cements where sufficient calcium has been removed, shifting relative concentration of remaining Mg towards dolomite as observed in the adjacent aragonite/dolomite cement bands associated with cyanobacterial activity.

Endolithic cyanobacterial activity is also the most likely explanation for the parallel dolomite and aragonite bands observed in all samples. These bands formed along discontinuities similar to regrowth or conceptacle bands and both these types of crustal weakness of increased porosity allow penetration of cyanobacteria into the crust. Previous research^49^ demonstrated that calcium mobilized by the *Mastigocoleus* cyanobacteria may precipitate on the substrate surface as aragonite. It seems probable that as cyanobacteria remove calcium from the Mg-C skeleton^49^, it is re-precipitated as aragonite behind the cyanobacterial filament, and the skeleton at the cyanobacterial front is transformed towards dolomite. For the greenhouse samples, the accelerated rate of CCA bioerosion^21^ probably created porosity that allowed undersaturated seawater to penetrate and either wash out the mobilized calcium and/or undersaturation prevented aragonite reprecipitation. Conversely, the higher seawater carbonate saturation state (Supplementary Table 1) and colder temperature of the icehouse conditions, would have slowed bacterial erosion rates and enhanced abiotic aragonite precipitation where it did occur. This could explain the increase of aragonite in icehouse samples.

Our study reconciles the different roles of pCO_2_/temperature and Mg:Ca ratios in controlling net CCA mineralogy as follows. Our results and previous research show that skeletal minerals under the direct control of living tissue change little with oscillations of pCO_2_ (Fig. 2, surface layer and Ries^12^), but experience considerable changes with Mg:Ca^9^. In contrast to this, post-depositional changes to CCA mineralogy, such as infilling and alteration of the nonpigmented crust, are driven by temperature and pCO_2_ variability, supporting Sandberg’s^10^ study concluding a correlation of temperature, CO_2_ and carbonate mineral changes.

Finally, while dolomite has been suggested to confer some degree of resistance for CCA skeletons to chemical dissolution under short-term ocean acidification conditions^22^, here we demonstrate that through resistance to erosion by endolithic algae (and thus retaining density), dolomite plays yet another role in decelerating net skeletal break-up. Dolomite occurs in greater abundance in the layers below the photosynthetic tissue; hence, protection against dissolution in pigmented layers may be limited in a high CO_2_ ocean, although pigmented organic coatings are known to protect CCA skeletons from dissolution in undersaturated seawater conditions^25^,^29^. As CO_2_ rises, chemical dissolution is likely to play a greater role than bioerosion, but this could change as temperatures increase. Furthermore, because the dolomite mineral is much less soluble in seawater than the high-Mg-calcite typically deposited by the coralline algae, CCA with dolomite may experience differential preservation and retain growth substrate compared with other species as CO_2_ rises. This process may be more important in shallow wave-swept environments where dolomite is more common. However, the combination of increased dissolution (associated with undersaturation of seawater with respect to high-Mg-calcite) together with increases in endolithic algal bioerosion^21^, ultrastructural changes^29^ and enhanced CCA mortality^20^,^21^ under greenhouse conditions could compromise the physical stability of the CCA community, and ultimately, that of the reef. Understanding how these processes affect the mineralogy and physical structure of CCA in the open environment is a critical step to understanding how coral reef coralline algae will cope with ocean acidification and warming into the future, and will shed light on processes that drove comparable dolomite formations in the past.

## Methods

### Experimental setup and manipulations

The experiment was conducted in the outdoor flow-through aquaria facilities of Lizard Island Research Station (14°40′08′′S, 145°27′34′′E), northern Great Barrier Reef, Australia. The CO_2_ treatments included three CO_2_-dosing /pH regimes that represent a range of historical (pre-industrial, target value for CO_2_: 290 μatm and pH_SW_: 8.15), present-day (ambient control, 357 μatm and 8.09), and projected early 22nd century (high, 1,230 μatm, 7.63) CO_2_ levels under the representative concentration pathways 8.5 (RCP8.5) scenario^51^,^52^. The CO_2_ manipulations were conducted following methods described earlier^53^ with the exception that ambient control pH was not regulated. In short, for the high CO_2_ treatment, pH was regulated by injection of analytical grade CO_2_ into 200 l sumps and logged every 10 minutes using a pH sensor (Mettler Toledo, InPro4501VP). pCO_2_ in the pre-industrial treatment was reduced by filtering the air through a column of soda lime before injecting it into the water in the sump^53^. There were two temperature levels, ambient control (27.9 °C±1.55, mean±s.e.) and elevated (29.85 °C±1.73), achieved using one water heater (Aqua One, Glass Aquarium Heater, 100 W) per aquarium. There were five replicate 15 l aquaria per CO_2_ and temperature treatment combination, each containing three fragments of CCA. All tanks and fragments were randomly allocated to treatments using the random number function in Microsoft Excel. Possible issues associated with tank interdependence were addressed by using identically manufactured sumps across CO_2_ treatments, fed by the same water supply from the reef flat, and exposed to same light conditions. For mineralogy analyses, a subset of three aquaria (one fragment per aquarium) was used per treatment combination. A shade cloth was used over the aquaria to reduce the natural sunlight to approximately match the levels in native habitat (2–4 m). Average light level in the aquaria at 11 am was 220 μmol photons m^−2^ s^−1^. Each aquarium had a powerhead (Infinity 800, 25 w, Q800 l/h) with the outlet connected to a 20 × 1.8 cm plastic poly pipe with 6 4 mm diameter holes distributed along the pipe for water movement. The experiment ran for 2 months during October–December 2009.

### Specimen preparation

We used fragments of the CCA *P. onkodes* of c.a. 3 × 3 cm (3–6 mm thick) collected from the shallow (2–4 m depth) coral reefs of Lizard Island and prepared accordingly to methods described in Diaz-Pulido *et al.*^21^ CCA were acclimatized in tanks with running seawater during 15 days prior to the experiment. Fragments were then placed in the aquaria and continuously supplied with experimental water with a flow of 36 l h^−1^. CCA growth rates were determined as differences in wet weight between the first and last day of experiment. CCA samples were dried at 60 °C for 24 h at the end of the experiment to determine the relationship between wet and dry weight (regression analysis: *r*^2^=0.991, *n*=115, *P*<0.001). Bulk samples (mostly unpigmented skeleton) of CCA were prepared by breaking a small fragment off the crust, while surface scrapings (skeletons covered with photosynthetically active tissue) were taken by gently scraping the pink surface with a razor, taking care not to dig into the white crust. Pulse-amplitude modulation (PAM, model: Diving-PAM with red measuring light LED, 655 nm) fluorometry measurements of maximum quantum yield (settings: damp=2 and gain=3) were conducted on dark adapted crusts in subsequent experiments to confirm the lack of photosynthetic activity by CCA in the unpigmented skeleton.

### Mineralogy analyses

Powder XRD and scanning electron microscopy-energy dispersive spectroscopy (SEM-EDS) analyses were used to compare the mineralogy of skeletons covered by pigmented (pink) tissue (surface scrapings) versus that of skeletons mostly devoid of pink CCA tissue (bulk sample), among treatments. SEM-EDS was carried out using an Hitachi 4300-SE, equipped with an integrated Oxford X-Max element detector, operated at 15.0 kV, 25 mm working distance, current 0.6 nano ampere, beam interaction volume ~3 μm. Imaging was at 10 mm working distance and 10.0 kV. Samples were carbon coated. A sample from each of the pre-industrial 28 °C and high CO_2_ 30 °C treatment was embedded in resin and polished for precise SEM-EDS measurements (Fig. 4a,b,d–f), other samples were fractured (Figs 4c and 5a–c). XRD was carried out using a SIEMENS D501 Bragg-Brentano diffractometer equipped with a graphite monochromator and scintillation detector, using CuK radiation. The precision for the quantification of carbonates using XRD data from instrument D501 with the given run conditions is ~0.5%, therefore, the quantified mineral amounts and differences among treatments are well above the detection level. Scan interpretation followed procedures describe earlier^5^,^54^. XRD and SEM-EDS analyses were conducted at The Australian National University. Aragonite was quantified using the area under the curve method (see below for details and calibrations) and the Mg-content of calcite was calculated from the (104) peak position as described earlier^5^. Comparisons of peak asymmetry due to dolomite were based on the asymmetrical mol% method as developed by Nash *et al*.^54^ (Supplementary Figs 2 and 3), while mineral quantities were calculated using the Rietica programme for Rietveld refinement^55^ (see below for details, Supplementary Tables 6–8). Inductively coupled plasma (ICP)-atomic emission spectroscopy (AES) was undertaken to calibrate calculated mineral quantities (Supplementary Tables 7 and 8). The dolomite range is as determined by Zhang *et al.*^35^ for disordered dolomite and Nash *et al*.^54^ for dolomite in CCA. In this study, we use the term ‘dolomite’ to refer to magnesium composition in the range 38–62 mol % MgCO_3_ (as per Nash *et al*.^5^) without inferring cation-ordering status, that is, regardless of whether the dolomite is ordered, or partially ordered or completed disordered (‘protodolomite’).

### Initial identification of dolomite using asymmetry %

Previous work has demonstrated that the asymmetry often observed for the XRD Mg-calcite (104) peak indicates the presence of dolomite^5^,^22^,^54^. Simple techniques have been developed to quantify the relative extent of asymmetry^22^,^54^, as an indicator for the relative changes in dolomite content. In principle, this technique uses the mol% MgCO_3_ calculated from the *d*-value for the highest point of the Mg-calcite peak position and compares this with the calculated mol% for the *d*-value at the peak position identified using the area under the curve function in scan processing software to return the gravity centre of the curve. The asymmetry % on the *y* axis for Fig. 2 is a numerical representation of the extent of asymmetry on the XRD Mg-calcite peak. A symmetrical peak shape indicates that only Mg-calcite is present, while curve asymmetry towards high 2-theta indicates the presence of more magnesium rich Mg-calcite (Supplementary Fig. 2) and strong asymmetry over the dolomite position indicates dolomite. Usually, the calculation of the mol% MgCO_3_ is performed by taking the *d*-value at the highest point of the Mg-calcite curve and using this in a calibration equation. This is the method used to calculate the mol% MgCO_3_ in Fig. 2c. However, for curves with asymmetry, reporting only the mol% does not provide information on varying curve shape, which can also indicate the presence of other carbonate minerals (low-Mg-calcite will have asymmetry towards the low 2-theta side). The scan processing programme we use (EVA Diffrac Plus) has an area function that returns the gravity centre peak for the entire curve (using an average for each measurement position—weighted by its peak intensity) (Supplementary Fig. 2). This incorporates the influence of the asymmetry, and the peak position is shifted towards higher 2-theta when higher Mg-calcite, dolomite and/or magnesite are present (or lower 2-theta if low-Mg calcite present). Using the *d*-value from the gravity centre peak in the calibration equation returns a higher mol% MgCO_3_ than using the highest point of the curve when there is measurable asymmetry. We subtract the standard mol% MgCO_3_ from the asymmetry mol% MgCO_3_ and use this difference to compare the various asymmetries (and therefore presence or absence of dolomite) across samples. This method is developed further in Nash *et al.*^54^. CCA samples were not bleached or chemically cleaned prior to XRD, as this can lead to reduced dolomite asymmetry^5^.

### Identifying multiple dolomite minerals and composition

The composition of dolomite was determined using a simple curve subtraction technique^54^, which relies on subtracting a symmetrical curve from the Mg-calcite curve to isolate the remnant asymmetry (Supplementary Fig. 3a,b). The process is repeated for the remnant asymmetrical curve to isolate the portions that are in the higher Mg-calcite and dolomite ranges. This allows a first estimation of the Mg-rich carbonates present. This process identified a higher Mg-calcite (H-HMC) in both the control (26.7 mol% MgCO_3_) and dolomite enriched high CO_2_/high temperature CCA (28.7 mol% MgCO_3_) and three dolomite phases. The dolomite for the ambient control was 33.1 mol% (~39–44 mol% disordered dolomite) compared with the higher Mg-content for the greenhouse CCA of 40.9 mol% (52–56 mol% disordered dolomite). Furthermore, the greenhouse CCA had an additional dolomite phase (Dol 2) of 52 mol% (disordered dolomite value not available as this is beyond the range of calibration^35^). These initial estimates were then used to create dolomite entries for Rietveld refinement using the Rietica programme^55^. Refinements were undertaken using the greenhouse CCA. Unit cell parameters for these phases were estimated from information in Zhang *et al.*^35^ The unit cell parameters were allowed to vary with the refinement to obtain best fit, always ensuring that the results were still representative of the compositional range modelled. The best fit was obtained by adding an additional Mg-carbonate phase at the high end of the dolomite range (~60 mol% for ordered dolomite) (Supplementary Table 6). The final refined peak positions when used to calculated mol% MgCO_3_ resulted in compositions that were regularly spaced from the H-HMC to the third dolomite (Supplementary Fig. 3c), suggesting that these may have been average compositions for a continuum of Mg compositions over that range, rather than isolated phases with only that MgCO_3_ composition. This is consistent with the range of dolomite compositions measured by SEM-EDS (this paper and Nash *et al.*^5^).

### Quantification of aragonite

Rietveld refinement for quantification was undertaken using the above identified mineral phases. Aragonite calibrations were run with 3 and 10% of bleached coral (aragonite) and 97 and 90% of CCA, respectively. The closest match was found by using the area under the aragonite curve (peak 1 and 2) as a percentage of the total area under the curves of aragonite and the HMC peak (including the asymmetrical portion). Quantification by Rietveld refinement returned nearly double the spiked amount (aragonite added: 3%, substation quantification: 3 to 3.5%, Rietica quantification: 5.8%, Rietica Chi^2^: 1.38; aragonite added: 10, subtraction quantification: 10.8 to 11.3%, Rietica quantification: 22.00%, Rietica Chi^2^: 1.83). Therefore aragonite quantities used herein were determined using the area under the curve method. There are a few reasons why the Rietveld refinement could be returning these higher quantifications. As observed in SEM, the aragonite crystals were well crystallized with the typical aragonite needles or blocky needles, whereas there was a range of morphologies for the dolomite composition, from the fine spheroidal dolomite, larger spheroidal dolomite, transformation of entire skeleton (crystal morphology not detectable), Mg-calcite with calcium removed and other work has documented rhomb dolomite in the cell lining and fine precipitate in conceptacles^22^. These small and poorly crystallized dolomites would reflect X-rays less strongly than well crystallized minerals and thus the XRD peak intensity would be reduced. As Rietveld refinement models peak intensity by varying mineral abundance, this could result in overestimation of the well crystallized aragonite compared with the poorly crystallized dolomite. Another possibility is that there is substantial amorphous content in the CCA, that is, uncrystallized mineral, which would not diffract X-rays, but relatively dilute all minerals present. While there may be some amorphous mineral, there was no clear amorphous hump on the XRD pattern, suggesting that any amorphous material would be in minor amounts and thus could not account for a doubling of the spiked phase (aragonite). Using biogenic carbonates for quantification calibration may be problematic due to these variations in crystal size and possibility of amorphous material; however, as our work here is with biogenic carbonates, these were considered the most representative to use for the calibration of aragonite peak intensity.

### Quantification of Mg-Calcite and dolomite

Rietveld refinement using Rietica was undertaken for quantification. Unit cell parameters were fixed for the different dolomite phases and allowed to vary for the Mg-calcite and H-HMC peaks. Quantities obtained were then normalized to 100% without aragonite (Supplementary Table 3). The aragonite quantities determined using the area under the curve were added and other mineral phases renormalized (Supplementary Table 4). This was considered to be the best estimate for mineral phases present.

### Calibration of dolomite quantities

ICP-AES was undertaken to measure bulk magnesium concentration and for calibration of dolomite quantities. ICP-AES followed methods in ref. 5. The best calibration was found using 17.5 mol% (Mg-calcite), 27–30 mol% (H-HMC), 47 mol% (Dol 1), 55 mol% (Dol 2), 65 mol% (Dol 3) and 95 mol% (beyond dolomite—magnesite) (Supplementary Tables 7 and 8). Average difference for the calculated bulk Mg as per Rietica quantification compared with the ICP-AES bulk Mg was 0.3 mol%, range; −1.8 to 2.7 mol%.

### Comment on precision and accuracy of quantification method

As it is impossible to physically separate the Mg-calcite and dolomite, the quantification cannot be independently verified. The Rietveld refinement method is well established and even though the absolute quantities should be considered a best estimate, the calculated differences are a mathematical difference in area under the relevant curves and therefore, the relative increase in amount of dolomite phases in the greenhouse is mathematically reliable. Further, these results are supported by the ICP-AES data for the greenhouse samples.

### Data analysis

Data for aragonite %, asymmetry % (dolomite) and mol% MgCO_3_ were analysed using multifactorial analyses of variance with pCO_2_, temperature and layer treatments as fixed factors and aquaria (three CCA fragments) as replicates. Growth rates were analysed using a two-way nested ANOVA with pCO_2_ and temperature as fixed factors with CCA fragments nested within tanks. Since the nested component was not significant (*P*=0.53), growth data were pooled and fragments used as replicates^56^. Data were checked for outliers (stem-and-leaf plots), normality and homogeneity of variance (Cochran’s test). Data for % aragonite was log-transformed prior to ANOVA. When significant interactions occurred, we conducted individual ANOVAs within treatment combinations. *Post hoc* comparisons were examined using Tukey HSD test. Regression analyses were conducted using the least squares method to explore the relationship between CCA dissolution rates and dolomite relative abundance (% asymmetry and weight % dolomite calculated from the Rietica method, Supplementary Fig. 2). Data analyses were performed using SYSTAT software.

### Water chemistry analyses

Samples for water carbonate chemistry analyses were collected from the experimental aquaria every 4 h through a 24-h period in 11 December 2009 following protocols from Dickson *et al*.^57^ (Supplementary Table 1). Samples were also collected from the sumps but did not show differences compared with the experimental aquaria, indicating the physiological activity of CCA did not affect bulk water conditions. Total alkalinity was measured using a Metrohm auto-titrator at Edith Cowan University. Total alkalinity and pH(s-w scale) data were used to calculate pCO_2_, total carbon (TC), bicarbonate (HCO_3_), carbonate (CO_3_), saturation state of seawater (Ω) with respect to calcite (Ω_cal_) and aragonite (Ω_arag_) using the programme CO2sys ( http://www.cdiac.ornl.gov/oceans/co2rprt.html) using constants described earlier^21^ and salinity 35.3. Ω high-Mg-calcite (Ω_HMC_) was calculated for a 16.4%mol MgCO_3_ content as described in Diaz-Pulido *et al*.^21^ and Ω for disordered dolomite (Ω_dol-diso_) was determined using a solubility constant *K*_*D-disordered*_ 10^−16.62^ (at 28 °C) from Carpenter^58^ and PHREEQ ( http://www.ndsu.edu/pubweb/webphreeq/webphreeq-2.0/), following methods from Diaz-Pulido *et al*.^21^ Computation of Ω_dol-diso_ is problematic as discussed by Arvidson and Mackenzie^38^ and Burns *et al.*^59^, thus the calculated values should be regarded as approximate estimations, but nevertheless indicate that seawater from all treatment combinations was considerably supersaturated with respect to dolomite (Supplementary Table 1).

## Author contributions

G.D.-P. and M.C.N. conceived and designed the study, collected, processed and analysed the data, and wrote the manuscript. M.C.N. and U.T. performed XRD analysis and M.C.N. SEM examination. G.D.-P., C.R.-N. and D.B. maintained the experiments at Lizard Island and K.R.N.A. provided insight into the experimental methods. All authors contributed substantial comments and editorial revisions and contributed to the writing of the paper.

## Additional information

**How to cite this article**: Diaz-Pulido, G. *et al.* Greenhouse conditions induce mineralogical changes and dolomite accumulation in coralline algae on tropical reefs. *Nat. Commun.* 5:3310 doi: 10.1038/ncomms4310 (2014).

## Supplementary Material

Supplementary Information Supplementary Figures 1-3 and Supplementary Tables 1-8
