# Supplementary material for: Greenhouse conditions induce mineralogical changes and dolomite accumulation in coralline algae on tropical reefs
Source: Nat Commun. 2014 Feb 12;5:3310. doi: 10.1038/ncomms4310 (PMC3929803; doi:10.1038/ncomms4310)
Supplement: Supplementary Information — Supplementary Figures 1-3 and Supplementary Tables 1-8 [file ncomms4310-s1.pdf]

Supplementary Information

Supplementary Figures

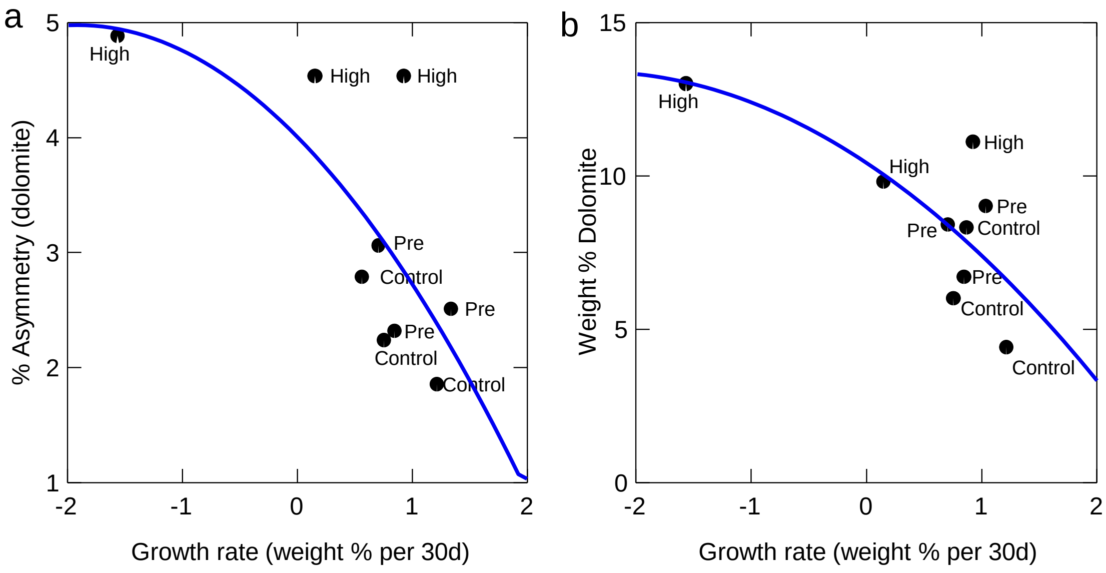

Supplementary Figure 1. Relationship between net growth rates of the crustose coralline alga (CCA) *Porolithon onkodes* and dolomite abundance in bulk skeletons under the high temperature treatment (30°C).

Dolomite abundance calculated accordingly to (**a**) asymmetry method and (**b**) Rietica method (weight %). CCA samples were exposed to three levels of pCO_2 (Pre = preindustrial, Control = ambient, and High) for a period of two months in Lizard Island, GBR. Net growth rates (positive indicate calcification and negative skeletal dissolution) were normalised to the initial weight of each fragment and presented as percentage of change per 30 days. Regression analyses show positive relation between dissolution and dolomite abundance (a, r^{2} = 0.48; *P* = 0.039; n=9 and b, r^{2} = 0.51; *P* = 0.031; n=9). A quadratic regression line is included as it seems to better represent the trends in the data.

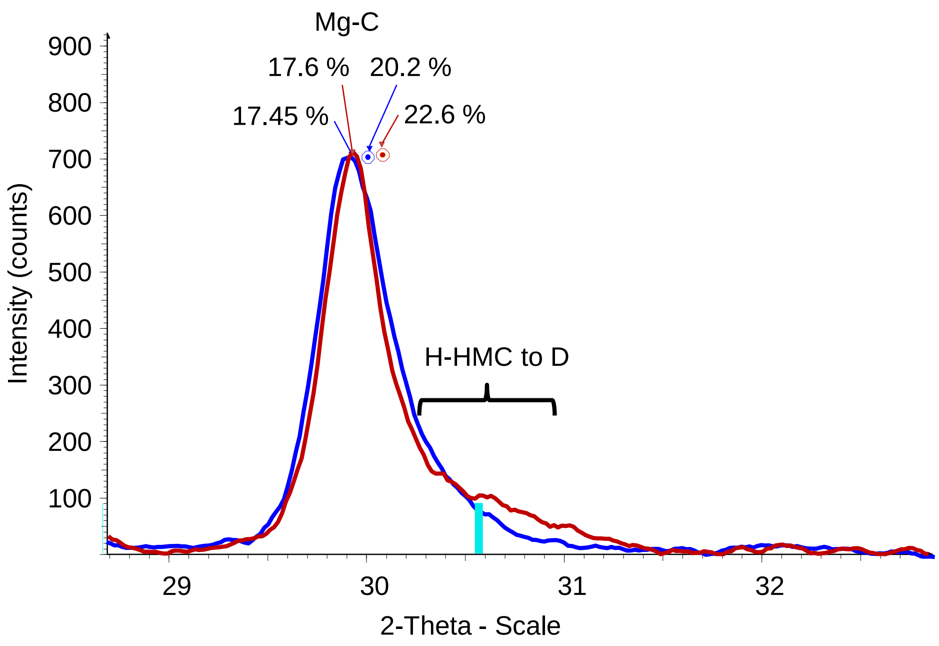

Supplementary Figure 2. Asymmetry and peak position.

Application of asymmetry calculations for High CO_2 and 30°C (red) (GD114 in Fig. 4 and 5 of main MS), and pre-industrial CO_2 and 28°C (blue) (GD2 in Fig. 4 of main MS). The peak position mol%'s for both are comparable 17.45 and 17.6, however once the asymmetry is incorporated then the visible difference in curve asymmetry over the Higher Mg-calcite (H-HMC) and dolomite (D) range is reflected in the higher mol% $MgCO_3$ (22.6) calculated for the High CO_2/30°C than for the pre-industrial/28° sample (20.2). For the asymmetry % in Fig. 2a (main MS) the peak mol% has been subtracted from the asymmetry mol% to give the net change due to asymmetry.

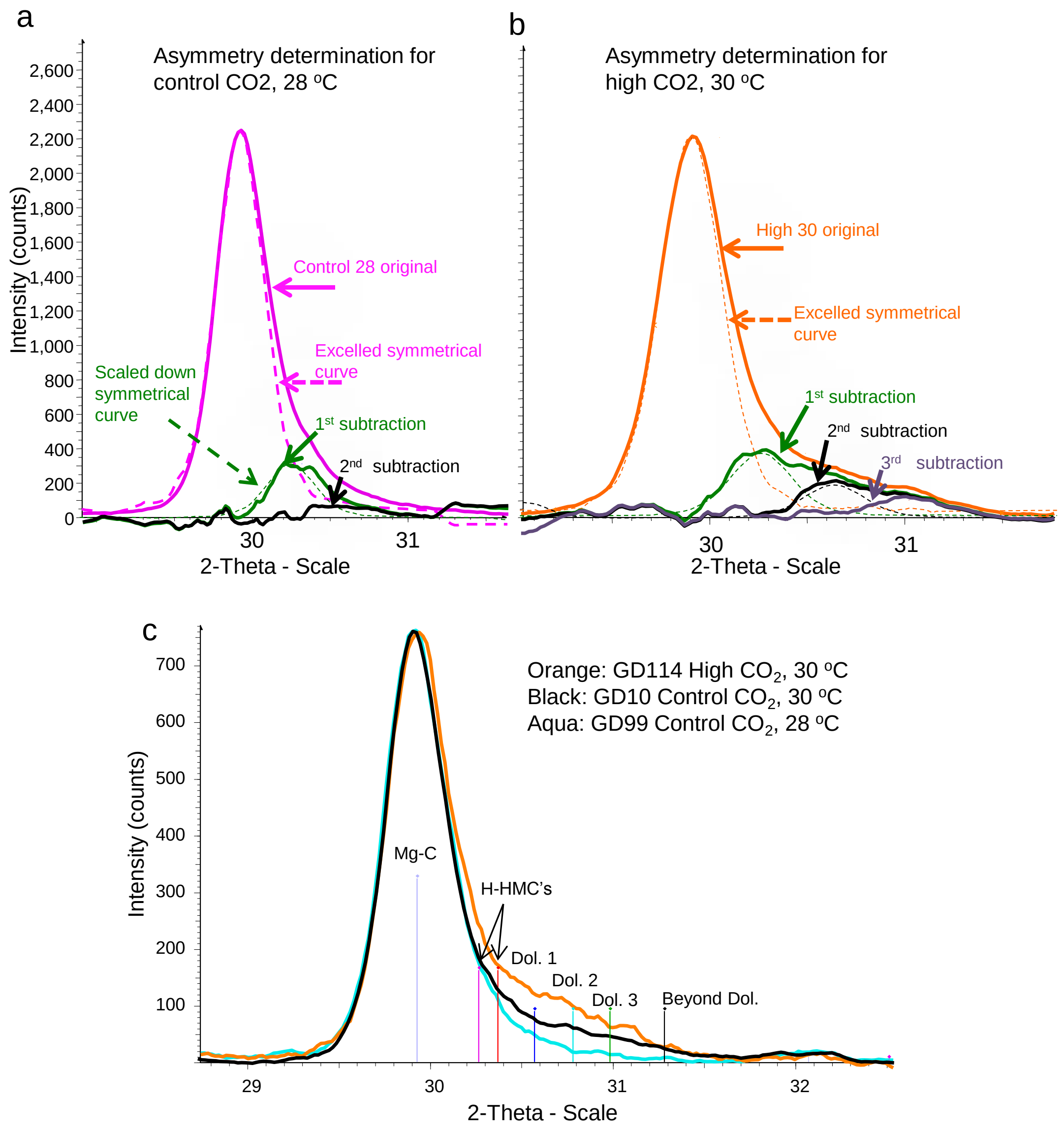

Supplementary Figure 3. Subtraction using a symmetrical curve to identify and quantify dolomite phases.

(a) Ambient control samples. (b) High CO_2 high temperature 30°C samples. Scans for each treatment were added and only the average is shown here. (c) Peak positions for refined mineral phases. Examples of scans overlaid for context.

Supplementary Tables

Supplementary Table 1. Carbonate chemistry parameters.

Treatment	T °C	pH-sw	pCO_2 µatm	TA µmol kg^{-1}	TC µmol kg^{-1}	HCO^-_3 µmol kg^{-1}	CO_3^{2-} µmol kg^{-1}	Ωcal	Ωarag	ΩHMc (16.4)	Ωdol-diso
Pre-industrial (8.15)	28	8.15 (0.06)	296 (57)	2398 (18)	1978 (57)	1675 (84)	296 (30)	7.14 (0.71)	4.76 (0.48)	1.73 (0.17)	151.82 (30.49)
	30	8.15 (0.06)	293 (57)	2402 (17)	1960 (55)	1642 (84)	311 (31)	7.54 (0.75)	5.06 (0.50)	1.88 (0.19)	188.41 (38)
Ambient - Control (8.09)	28	8.09 (0.03)	357 (35)	2401 (17)	2029 (31)	1754 (41)	265 (13)	6.40 (0.31)	4.26 (0.21)	1.56 (0.08)	121.25 (11.49)
	30	8.09 (0.03)	355 (33)	2406 (27)	2012 (32)	1724 (40)	280 (14)	6.78 (0.35)	4.55 (0.23)	1.69 (0.09)	151.22 (15.12)
High (7.63)	28	7.63 (0.02)	1233 (60)	2415 (21)	2285 (17)	2139 (15)	114 (5)	2.75 (0.13)	1.83 (0.09)	0.67 (0.03)	22.38 (2.074)
	30	7.64 (0.02)	1225 (77)	2420 (35)	2276 (37)	2123 (37)	123 (5)	2.97 (0.12)	1.99 (0.08)	0.74 (0.03)	29.07 (2.281)

T: temperature; pH: pH seawater scale; pCO_2: carbon dioxide partial pressure; TA: total alkalinity; TC: total carbon; HCO_3: bicarbonate; CO_3: carbonate; Ωcal: saturation state of seawater with respect to calcite; Ωarag: aragonite saturation state; ΩHMc: High-Magnesium calcite saturation state (for 16.4 %mol $MgCO_3$). Ωdol-diso: disordered dolomite saturation state. Values are means of 6 samples (standard deviation).

Supplementary Table 2. Analyses of variance (ANOVA) to test for the effects of CO_2, temperature and tissue layer on *Porolithon onkodes* mineralogy.

Source of variation	Df	MS	F	*P*	Conclusion (Tukey test)
Asymmetry (dolomite)					
CO_2	2	0.32	1.45	SL = 0.131;	
				BU & 28 = 0.817	
				BU & 30 < 0.001	High > Pre = Cont
Temperature (Temp)	1	1.46	6.72	SL = 0.139	
				BU & HCO_2 = 0.002	30 > 28
Layer	1	47.81	219.46	<0.001	BU > SL
CO_2*Temp	2	1.85	8.50	0.002	s
CO_2*Layer	2	1.94	8.90	0.001	s
Temp * Layer	1	0.09	0.40	0.532	ns
CO_2 *Temp * Layer	2	1.7	7.80	0.002	s
Error	24	0.22			
Aragonite (%, transf.)					
CO_2	2	0.47	16.02	30 = 0.568; 28=0.001	Pre > Cont = High
Temperature	1	0.59	19.90	Pre = 0.001	28 > 30
Layer	1	0.78	26.57	<0.001	BU > SL
CO_2*Temp	2	0.21	7.04	0.004	s
CO_2*Layer	2	0.03	1.0	0.383	ns
Temp * Layer	1	<0.01	0.05	0.830	ns
CO_2 *Temp * Layer	2	0.01	0.42	0.664	ns
Error	24	0.03			
$MgCO_3$					
CO_2	2	0.43	2.29	0.123	ns
Temperature	1	0.63	3.37	SL=0.004; BU=0.34	30 > 28
Layer	1	13.79	73.65	<0.001	BU > SL
CO_2*Temp	2	0.10	0.52	0.604	ns
CO_2*Layer	2	0.30	1.59	0.226	ns
Temp * Layer	1	1.96	10.47	0.004	s
CO_2 *Temp * Layer	2	0.22	1.19	0.322	ns
Error	24	0.19			

SL: surface layer; BU: Bulk tissue. HCO_2; High CO_2; Pre: pre-industrial. Df: degrees of freedom, MS: mean square, F: F-ratio.

Supplementary Table 3. Mineral quantities (weight %, normalized to aragonite) in bulk skeletons of *Porolithon onkodes*. Data presented for each CO_2 and temperature treatment.

CO_2	T°C	Sample	Mineral (weight %)						
			Mg-C	H-HMC	Dol 1	Dol 2	Dol 3	Total Dol	BD
Pre	28	GD25	88.5	4.3	7.2	0.0	0.0	7.2	0.0
Pre	28	GD69	89.4	3.1	6.0	0.8	0.6	7.4	0.0
Pre	28	GD2	88.5	4.3	7.2	0.0	0.0	7.2	0.0
		average	88.8	3.9	6.8	0.3	0.2	7.3	0.0
Pre	30	GD101	86.9	4.6	4.2	3.1	1.3	8.6	0.0
Pre	30	GD40	82.2	6.3	3.4	3.2	2.8	9.4	2.5
Pre	30	GD18	78.0	15.2	6.9	0.0	0.0	6.9	0.0
		average	82.3	8.7	4.8	2.1	1.3	8.2	0.8
Cont	28	GD62	65.5	23.0	11.5	0.0	0.0	11.5	0.0
Cont	28	GD99	83.5	12.5	4.0	0.0	0.0	4	0.0
Cont	28	GD49	81.8	6.3	3.2	2.3	2.6	8.1	3.9
		average	76.9	13.9	6.2	0.8	0.9	7.9	1.3
Cont	30	GD24	83.5	11.9	4.5	0.0	0.0	4.5	0.0
Cont	30	GD36	79.5	14.2	6.3	0.0	0.0	6.3	0.0
Cont	30	GD10	78.4	5.9	3.0	1.9	3.5	8.4	7.3
		average	80.5	10.7	4.6	0.6	1.2	6.4	2.4
High	28	GD37	83.1	13.0	3.9	0.0	0.0	3.9	0.0
High	28	GD81	84.8	12.2	3.0	0.0	0.0	3	0.0
High	28	GD7	81.5	13.8	4.7	0.0	0.0	4.7	0.0
		average	83.1	13.0	3.9	0.0	0.0	3.9	0.0
High	30	GD48	79.5	6.1	4.0	3.3	3.9	11.2	3.4
High	30	GD71	80.3	7.5	3.3	3.8	3.1	10.2	2.2
High	30	GD114	76.8	7.0	4.9	4.4	4.1	13.4	2.9
		average	78.8	6.9	4.0	3.8	3.7	11.5	2.8

Mg-C: Magnesium calcite; H-HMC: Higher high magnesium calcite. Dol: dolomite.

BD: beyond dolomite.

Supplementary Table 4. Mineral quantities (weight %) in bulk skeletons of *Porolithon onkodes*. Data presented for each CO_2 and temperature treatment and includes aragonite (Arag) from subtraction method.

CO_2	T^oC	Sample	Mineral (weight %)							
			Arag-Sub	Mg-C	H-HMC	Dol 1	Dol 2	Dol 3	Total Dol	BD
Pre	28	GD25	7.5	81.8	4.0	6.7	0.0	0.0	6.7	0.0
Pre	28	GD69	8.0	82.3	2.8	5.5	0.7	0.5	6.7	0.0
Pre	28	GD2	4.4	84.5	4.1	6.9	0.0	0.0	6.9	0.0
		average	6.7	82.9	3.7	6.4	0.2	0.2	6.8	0.0
Pre	30	GD101	1.4	85.6	4.5	4.1	3.1	1.3	8.5	0.0
Pre	30	GD40	1.5	80.9	6.2	3.3	3.1	2.7	9.1	2.4
Pre	30	GD18	1.4	76.9	15.0	6.8	0.0	0.0	6.8	0.0
		average	1.4	81.1	8.5	4.7	2.1	1.3	8.1	0.8
Cont	28	GD62	2.2	64.1	22.5	11.2	0.0	0.0	11.2	0.0
Cont	28	GD99	3.4	80.7	12.1	3.8	0.0	0.0	3.8	0.0
Cont	28	GD49	3.9	78.6	6.0	3.1	2.2	2.5	7.8	3.7
		average	3.1	74.5	13.5	6.1	0.7	0.8	7.6	1.2
Cont	30	GD24	1.8	82.0	11.7	4.5	0.0	0.0	4.5	0.0
Cont	30	GD36	2.7	77.4	13.8	6.1	0.0	0.0	6.1	0.0
Cont	30	GD10	0.0	78.4	5.9	3.0	1.9	3.5	8.4	7.3
		average	1.5	79.3	10.5	4.5	0.6	1.2	6.3	2.4
High	28	GD37	0.0	83.1	13.0	3.9	0.0	0.0	3.9	0.0
High	28	GD81	1.5	83.5	12.0	3.0	0.0	0.0	3	0.0
High	28	GD7	1.6	80.2	13.5	4.7	0.0	0.0	4.7	0.0
		average	1.0	82.3	12.9	3.8	0.0	0.0	3.8	0.0
High	30	GD48	0.0	79.5	6.1	4.0	3.3	3.9	11.2	3.4
High	30	GD71	1.5	79.1	7.4	3.2	3.7	3.0	9.9	2.1
High	30	GD114	0.9	76.1	6.9	4.8	4.3	4.0	13.1	2.9
		average	0.8	78.2	6.8	4.0	3.8	3.6	11.4	2.8

Mg-C: Magnesium calcite; H-HMC: Higher magnesium calcite. Dol: dolomite. BD: beyond dolomite.

Supplementary Table 5. Analyses of variance (ANOVA) to test for the effects of CO_2 and temperature on the relative mineral quantities in bulk skeletons of *Porolithon onkodes*. Data from Supplementary Table 4.

Source of variation	Df	MS	F	*P*	Conclusion (Tukey test)
Dolomite (%)					
CO_2	2	0.71	0.191	Within 28°C = 0.172	ns
				Within 30°C = 0.023	High > Cont
Temperature (Temp)	1	29.13	7.83	Within Pre = 0.119	ns
				Within Cont = 0.628	ns
				Within High = 0.002	30 > 28
CO_2*Temp	2	30.60	8.23	0.006	s
Error	12	3.72			
Aragonite (%, transf.)					
CO_2	2	0.22	6.28	0.014	Pre > High
Temperature	1	0.33	9.25	0.010	28 > 30
CO_2*Temp	2	0.07	2.07	0.168	ns
Error	12	0.04			
$MgCO_3$					
CO_2	2	40.86	2.13	0.162	ns
Temperature	1	0.47	0.02	0.879	ns
CO_2*Temp	2	31.50	1.64	0.235	ns
Error	12	19.21			

HCO_2; Pre: pre-industrial CO_2; Cont: control CO_2; High: High CO_2.

Supplementary Table 6. Unit cell parameters after refinement in Rietica.

	Unit cell parameters			Mol % $MgCO_3$	
	a	C	*d*-space	Ordered	Disordered
Mg-C	4.91906	16.7439	2.986	17.5	Not applicable
H-HMC - lower	4.87711	16.5222	2.953	28.5	~32
H-HMC - upper	4.869	16.44	2.9428	32	~41 (range ~35.6-48.5)
Dolomite 1	4.842	16.32	2.9247	38	~48 (range ~40-55)
Dolomite 2	4.826	16.16	2.9045	44.8	> ~54
Dolomite 3	4.81	16.01	2.8863	50.9	no value available
Beyond Dolomite	4.7482	15.914	2.8587	60.1	no value available

Disordered compositions estimated from Zhang et al. [35].

Supplementary Table 7. Calibration using magnesite composition for Dolomite 3 and beyond dolomite. This is the closest calibration for the samples containing an apparent magnesite shoulder (and magnesite confirmed by SEM), however appears to result in an overestimation of Mg for the High CO_2, 30°C samples. Overall average difference (diff) = 0.38%.

CO_2	T^oC	Sample	mol% $MgCO_3$								
			17.5%	30.0%	47.0%	55.0%	95.0%	95.0%			
			HMC	H-HMC	Dol1	Dol2	Dol3	BD	calc Mg	ICP Mg	diff
Pre	28	GD25	14.3	1.2	3.1	0.0	0.0	0.0	18.7	20.3	-1.6
Pre	28	GD69	14.4	0.9	2.6	0.4	0.5	0.0	18.8	20.2	-1.4
Pre	28	GD2	14.8	1.2	3.3	0.0	0.0	0.0	19.3	18.7	0.6
Pre	30	GD101	15.0	1.3	1.9	1.7	1.2	0.0	21.2	19.8	1.4
Pre	30	GD40	14.2	1.8	1.6	1.7	2.6	2.3	24.2	25.1	-0.9
Pre	30	GD18	13.5	4.5	3.2	0.0	0.0	0.0	21.1	18.0	3.1
Cont	28	GD62	11.2	6.7	5.3	0.0	0.0	0.0	23.2	22.4	0.8
Cont	28	GD99	14.1	3.6	1.8	0.0	0.0	0.0	19.6	17.1	2.5
Cont	28	GD49	13.8	1.8	1.5	1.2	2.4	3.5	24.2	27.0	-2.8
Cont	30	GD24	14.4	3.5	2.1	0.0	0.0	0.0	20.0	18.9	1.1
Cont	30	GD36	13.5	4.1	2.9	0.0	0.0	0.0	20.5	20.0	0.5
Cont	30	GD10	13.7	1.8	1.4	1.0	3.3	7.0	28.2	29.8	-1.6
High	28	GD37	14.5	3.9	1.8	0.0	0.0	0.0	20.3	21.3	-1.0
High	28	GD81	subsample not run for ICP-AES								
High	28	GD7	14.0	4.1	2.2	0.0	0.0	0.0	20.3	18.8	1.5
High	30	GD48	13.9	1.8	1.9	1.8	3.7	3.2	26.3	23.7	2.6
High	30	GD71	13.8	2.2	1.5	2.1	2.9	2.0	24.5	23.4	1.1
High	30	GD114	13.3	2.1	2.3	2.4	3.8	2.8	26.6	26.0	0.6

HMC: High magnesium calcite; H-HMC: Higher magnesium calcite. Dol: dolomite. BD: beyond dolomite. Calc Mg: calculated bulk Mg as per Rietica quantification. ICP Mg: bulk Mg concentration as per Inductively Coupled Plasma – Atomic Emission Spectroscopy.

Supplementary Table 8. Calibration using 65 mol% for dolomite 3 with magnesite samples excluded. The average difference (diff) is 0.3 mol%. Overall average difference = 0.26%.

CO_2	T°C	Sample	mol% MgCO3								
			17.5%	27.0%	47.0%	55.0%	65.0%	95.0%			
			HMC	H-HMC	Dol1	Dol2	Dol3	BD	calc Mg	ICP Mg	diff
Pre	28	GD25	14.3	1.1	3.1	0.0	0.0	0.0	18.5	20.3	-1.8
Pre	28	GD69	14.4	0.8	2.6	0.4	0.3	0.0	18.5	20.2	-1.7
Pre	28	GD2	14.8	1.1	3.3	0.0	0.0	0.0	19.2	18.7	0.5
Pre	30	GD101	15.0	1.2	1.9	1.7	0.8	0.0	20.6	19.8	0.8
Pre	30	GD40	n/d	n/d	n/d	n/d	n/d	n/d	n/d	n/d	n/d
Pre	30	GD18	13.5	4.0	3.2	0.0	0.0	0.0	20.7	18.0	2.7
Cont	28	GD62	11.2	6.1	5.3	0.0	0.0	0.0	22.6	22.4	0.2
Cont	28	GD99	14.1	3.3	1.8	0.0	0.0	0.0	19.2	17.1	2.1
Cont	28	GD49	n/d	n/d	n/d	n/d	n/d	n/d	n/d	n/d	n/d
Cont	30	GD24	14.4	3.2	2.1	0.0	0.0	0.0	19.6	18.9	0.7
Cont	30	GD36	13.5	3.7	2.9	0.0	0.0	0.0	20.1	20.0	0.1
Cont	30	GD10	n/d	n/d	n/d	n/d	n/d	n/d	n/d	n/d	n/d
High	28	GD37	14.5	3.5	1.8	0.0	0.0	0.0	19.9	21.3	-1.4
High	28	GD81	subsample not run for ICP-AES								
High	28	GD7	14.0	3.7	2.2	0.0	0.0	0.0	19.9	18.8	1.1
High	30	GD48	13.9	1.6	1.9	1.8	2.5	3.2	24.9	23.7	1.2
High	30	GD71	13.8	2.0	1.5	2.1	2.0	2.0	23.4	23.4	0.0
High	30	GD114	13.3	1.9	2.3	2.4	2.6	2.8	25.2	26.0	-0.8

n/d = no data. HMC: High magnesium calcite; H-HMC: Higher magnesium calcite. Dol: dolomite. BD: beyond dolomite. Calc Mg: calculated bulk Mg as per Rietica quantification. ICP Mg: bulk Mg concentration as per Inductively Coupled Plasma – Atomic Emission Spectroscopy.
